# Comparison of the performance of a chemiluminescence assay and an ELISA for detection of anti-GBM antibodies

**DOI:** 10.1080/0886022X.2019.1702056

**Published:** 2019-12-29

**Authors:** Ying Tan, Wei Pang, Xiaoyu Jia, Ming-hui Zhao

**Affiliations:** a Renal Division, Department of Medicine, Peking University First Hospital, Beijing, PR. China; b Institute of Nephrology, Peking University, Beijing, PR. China; c Key Laboratory of Renal Disease, Ministry of Health of China, Beijing, PR China; d Key Laboratory of Chronic Kidney Disease Prevention and Treatment, Ministry of Education, Beijing, China; e Peking-Tsinghua Center for Life Sciences, Beijing, PR China

**Keywords:** Autoantibodies, GBM, Goodpasture’s syndrome, Rapidly progressive crescentic glomerulonephritis (RPGN)

## Abstract

**Objective:**

Autoantibodies to the α3 chain noncollagen 1 domain of type IV collagen (α3(IV)NC1) are a serological hallmark in the diagnosis of anti-glomerular basement membrane (GBM) disease. The objective of our study was to compare the performance of anti-glomerular basement membrane (GBM) antibody detection by chemiluminescence immunoassay (CIA) and by enzyme-linked immunosorbent assays (ELISAs).

**Methods:**

Sera from outpatients who were suspected to have anti-GBM disease and 31 patients with biopsy-proven anti-GBM disease were collected. Thirty normal controls were also included. All samples were tested for anti-GBM antibodies by CIA and commercial ELISA. The anti-GBM antibody-positive samples were confirmed by a homemade ELISA coated with recombinant human α3(IV)NC1.

**Results:**

Compared with detection of anti-GBM antibodies with ELISA, detection of anti-GBM antibodies with CIA showed a positivity agreement of 70% and a negativity agreement of 98.6%. Among the 4 patients with different results, the anti-GBM antibody detection by CIA was in agreement with the homemade ELISA coated with recombinant human α3(IV)NC1 and the clinical diagnosis. In 31 patients with anti-GBM disease, good agreement was achieved in the detection of anti-GBM antibodies with CIA, commercial ELISA and the homemade ELISA (100%, 100%). The AUC for CIA and commercial ELISA was 0.987 and 0.966, respectively.

**Conclusions:**

The detection of anti-GBM antibodies with CIA demonstrated good sensitivity and specificity and was in good agreement with our homemade ELISA, which seems better than the commercial ELISA in suspected anti-GBM disease patients. The three assays performed in parallel in the diagnosis of anti-GBM disease patients.

## Introduction

Anti–glomerular basement membrane (anti-GBM) disease is a rare autoimmune disorder that is characterized by the production of autoantibodies directed to the GBM, rapidly progressive glomerulonephritis, and a high risk for alveolar hemorrhage [1]. Anti-GBM disease is a rare disease with a yearly incidence of 0.5–1 cases per million inhabitants, which can result in a rapid deterioration in renal function. The pathogenic role of anti-GBM antibodies has been demonstrated by their ability to transfer the disease to monkeys and by the recurrence of disease in human kidney allografts [2]. Early and accurate identification of anti-GBM antibodies are essential for renal prognosis since patients showed poor outcomes when the initial serum creatinine level was over 6.8** **mg/dL [3].

The target autoantigen of anti-GBM antibodies has been identified as the noncollagen domain 1 of the α3 chain of type IV collagen [α3(IV)NC1], with two major cryptic epitopes, EA and EB [4]. The confirmative feature of anti-GBM disease is the exhibition of linear deposits of immunoglobulin G (IgG) along the GBM on renal biopsy and detectable circulating antibodies against GBM by means of commercial enzyme-linked immunosorbent assay (ELISA) kits. However, there have been some cases reported with anti-GBM antibody deposition along the GBM but without positive findings for the autoantibodies by ELISA [5]. For these patients, diagnosis was difficult to confirm, and the indicator for plasmapheresis was deficient. Sometimes, the low sensitivity of anti-GBM detection may mislead the cessation of plasmapheresis even though anti-GBM antibodies are still not fully removed. Additionally, some patients are positive for anti-GBM antibodies by ELISA, but their autoantibodies do not recognize recombinant human α3 or demonstrate linear deposition in the GBM, which indicates false positive results that might partly be caused by the difference in coated bovine α3(IV)NC1 from the human homolog (unpublished data). Thus, more sensitive and specific methods need to be discovered.

A previous study demonstrated that the performance characteristics of an anti-GBM chemiluminescence immunoassay (CIA) had good sensitivity and specificity and were in good agreement with other methods [6]. Moreover, the CIA method takes 30** **min for one sample and 1** **min for the next sample.

The objective of this study was to compare the performance of an anti-GBM antibody CIA with an ELISA for clinical use.

## Methods

### Patients

Sera from patients who were suspected to have anti-GBM disease were collected at the Institute of Nephrology, Peking University from 5 June 2017 to 16 June 2017. The exclusion criteria were when the samples showed signs of hemolysis, lipemia, or bilirubinemia. Sera from 31 patients with biopsy-proven anti-GBM disease were collected from the Institute of Nephrology, Peking University from 2008–2015 and were preserved at –20 °C until use. Informed consent was obtained from each patient.

### Preparation of recombinant human α3(IV)NC1

Recombinant proteins were produced as described previously [7]. Briefly, cDNA from the NC1 domain of human type IV collagen α3 (Supplementary table 1) was ligated to a type X collagen triple-helix leader sequence and subcloned into the pcDNA3 vector. The constructs were then stably transfected into a human embryonic kidney (HEK 293) cell line, and recombinant proteins were harvested and purified from the medium and designated rα3.

### Anti-GBM assays

Polystyrene microtiter plates (Nunc Immunoplate, Roskilde, Denmark) were coated with 100** **μL of α3(IV)NC1 in coating buffer (50** **mM sodium carbonate [pH 9.6]) overnight at room temperature. rα3 was coated at 0.5** **μg/mL. The plates were then washed three times. A total of 100** **μL of human sera diluted 1:100 in PBS was added to each well. The plates were incubated at room temperature for 1** **h; after washing, alkaline phosphatase–conjugated goat anti-human IgG (Fc specific; Sigma, St. Louis, MO, USA) diluted 1:10 000 was added. Incubation resumed for 1** **h. P-nitrophenyl phosphate (1** **mg/mL; Sigma) in substrate buffer (1** **M diethanolamine and 0.5** **mM MgCl_2_ [pH 9.8]) was used as a substrate, and color development was measured spectrophotometrically at 405** **nm. All assays were run in duplicate, and when standard errors >10% were found, samples were reanalyzed. Plasma from 30 healthy blood donors was used to build a cutoff value as the mean +2 SD. The binding of the known positive control serum was set as 100%, and the blank control with PBS was set as 0%. The bindings of the tested sera were expressed as a percentage of that of the known positive sample.

Anti-GBM antibodies were also detected by a commercial CIA kit (INOVA, San Diego, USA) on a BIO-FLASH instrument and by a commercial ELISA kit (EUROIMMUN, Lübeck, Germany) on a EUROIMMUN Analyzer.

### Precision studies

Precision of the CIA was verified by performing the required testing according to the Clinical and Laboratory Standards Institute (CLSI) guidelines. For the precision study, the within-run, between-day, between-run, and total precision were determined by running two aliquots of the precision samples twice a day in a random order, with a minimum of 2** **h between each run. The samples were run on the same instrument for each assay, and runs were repeated for at least 5** **days, according to CLSI guideline EP5-A2.

### Statistical analyses

SPSS 16.0 statistical software was employed for statistical analysis. Spearman correlation analysis was carried out to analyze the correlation between groups, and *p* values <.05 were considered significant. Quantitative data were expressed as the mean ± SD or median with range (minimum, maximum). Receiver operating characteristic (ROC) analysis was carried out to analyze the discrimination between different methods and the homemade ELISA.

## Results

### General data of patients

Sera from 154 patients with suspected anti-GBM disease were collected. No samples showed signs of hemolysis, lipemia, or bilirubinemia.

### Comparison of CIA and ELISA in the detection of anti-GBM antibodies in suspected anti-GBM disease

**Figure 1.**
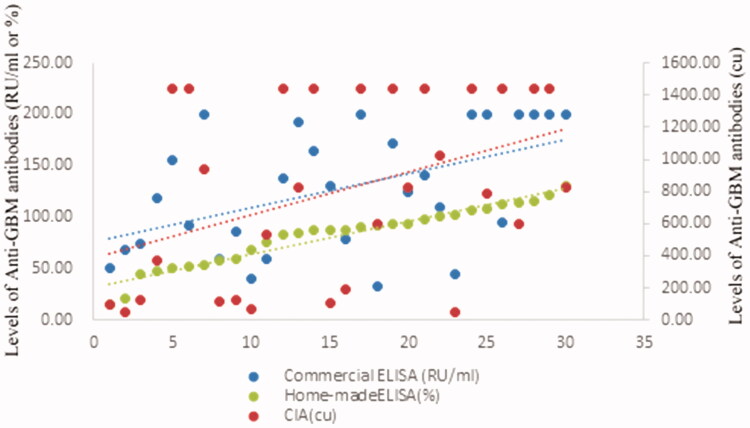
Scatter plot of levels of anti-GBM antibodies with different assays.

Compared with the detection of anti-GBM antibody with ELISA, the detection of anti-GBM antibody with CIA showed agreement of positivity of 63.6% and of negativity of 97.3% among the 154 outpatients (Figure 1).

Among the 4 patients with different results, the anti-GBM antibody detection by CIA was in agreement with the homemade ELISA coated with recombinant human α3(IV)NC1 and with the clinical diagnosis at a 2-year follow up (Table 1).

**Table 1.** The clinical features of patients with different results of anti-GBM antibody by ELISA and CIA.

No. of patients	Sex	Age	ELISA (RU/mL)	CIA (cu)	Home-made ELISA (%)	Renal biopsy	Clinical diagnosis
P1	M	50	Negative	42.1	47	Crescentic nephritis	Anti-GBM disease
P2	F	48	80.81	Negative	Negative	Minimal change disease	Nephrotic syndrome
P3	F	33	>200	Negative	Negative	Focal glomerulosclerosis	Chronic kidney disease
P4	F	65	26.02	Negative	Negative	Not performed	Latent nephritis

### Comparison of CIA and ELISA in the detection of anti-GBM antibodies in biopsy-proven anti-GBM disease

The general data of all 31 biopsy-proven anti-GBM disease patients are listed in Table 2. All the sera from the 31 patients with anti-GBM disease were tested by CIA, commercial ELISA and homemade ELISA (Table 2). The agreement of positivity and of negativity was both 100% for the commercial ELISA and the CIA.

**Table 2.** The clinical features of patients with biopsy-proven anti-GBM disease.

No.	Sex	Age (years)	Pathological diagnosis	ELISA (RU/mL)	CIA (cu)	Home-made ELISA (%)
1	M	75	Membranous nephropathy (Stage II) combined with Type I Crescentic nephritis	86	120.4	60
2	F	46	Membranous nephropathy (Stage II) combined with Type I Crescentic nephritis	33	600.1	92
3	M	48	Type I Crescentic nephritis	119	371.9	47
4	M	58	Type I Crescentic nephritis	338	>1437.8	83
5	F	20	Type I Crescentic nephritis	109	1021.2	101
6	F	58	Type I Crescentic nephritis	130	101.2	87
7	F	29	Type I Crescentic nephritis	40	67.3	68
8	M	50	Type I Crescentic nephritis	193	821.2	85
9	F	28	Type I Crescentic nephritis	>200	>1437.8	107
10	M	68	Type I Crescentic nephritis	74	125.2	45
11	F	41	Type I Crescentic nephritis combined with TMA	>200	593.6	114
12	F	49	Type I Crescentic nephritis combined with IgA nephropathy	59	113.2	58
13	F	73	Type I Crescentic nephritis	68	45.9	21
14	F	71	Type I Crescentic nephritis	>200	934.3	53
15	F	52	Type I Crescentic nephritis	>200	>1437.8	121
16	F	62	Type I Crescentic nephritis	95	>1437.8	113
17	M	60	Type I membranous nephropathy combined with Type I Crescentic nephritis	>200	>1437.8	115
18	M	20	Type I Crescentic nephritis	>200	820.4	131
19	M	58	Type I Crescentic nephritis	92	>1437.8	52
20	F	55	Type I Crescentic nephritis	>200	784.7	108
21	M	67	Type I + Type III Crescentic nephritis	60	529.1	75
22	F	49	Type I Crescentic nephritis combined with idiopathic memebraneous nephropathy	45	49.4	101.9
23	F	22	Type I Crescentic nephritis	155	>1437.8	50
24	M	48	Type I Crescentic nephritis	51	92	15
25	M	38	Type I Crescentic nephritis	165	>1437.8	87
26	M	17	Type I Crescentic nephritis	>200	>1437.8	90
27	F	35	Type I Crescentic nephritis	125	828	94
28	M	26	Type I Crescentic nephritis	79	191.7	87
29	M	27	Type I Crescentic nephritis	140	>1437.8	98
30	F	21	Type I Crescentic nephritis	172	>1437.8	94
31	M	34	Type I Crescentic nephritis	Neg	Neg	Neg

The levels of anti-GBM antibodies according to CIA were significantly correlated with those according to commercial ELISA (*r*** **=** **0.458, *p* =*** ***.001) and homemade ELISA (*r*** **=** **0.297, *p**** ***=*** ***.028).

### The prediction of levels of anti-GBM antibodies detected by CIA or commercial ELISA

**Figure 2.**
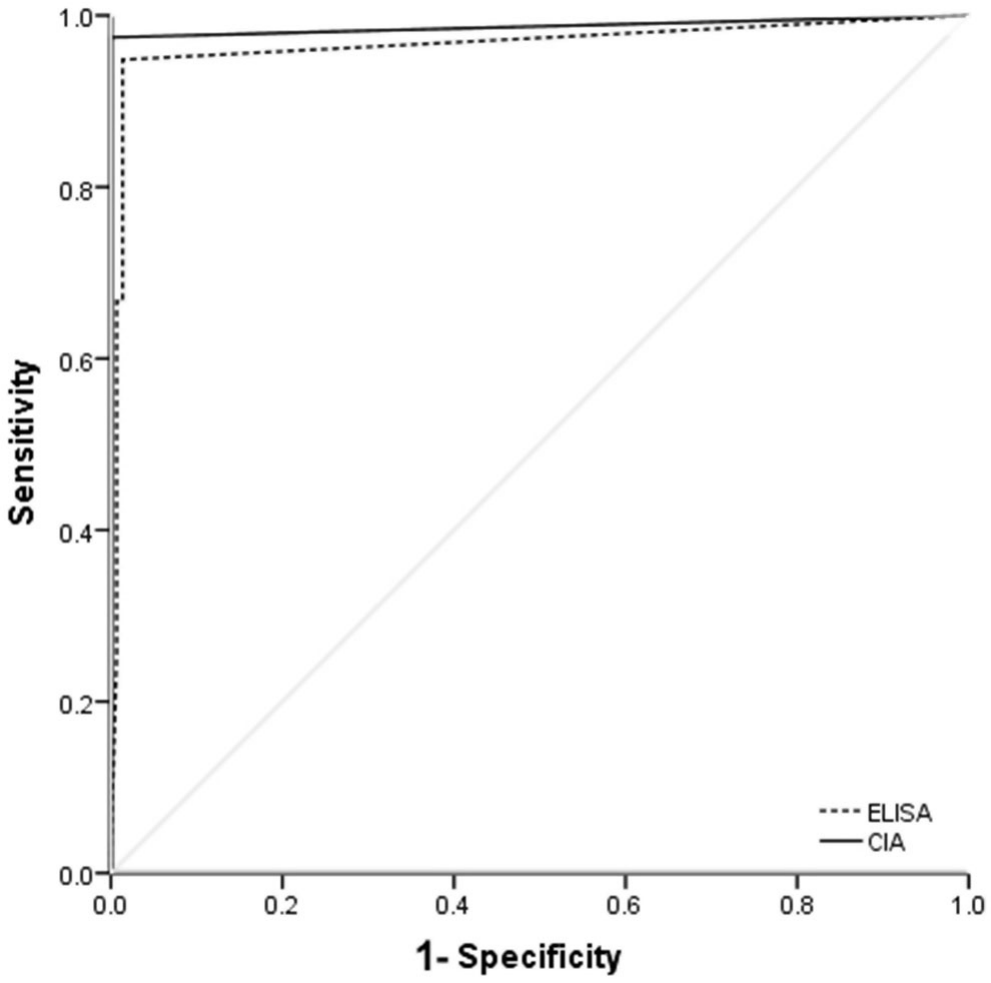
ROC curves for predicting anti-GBM disease based on commercial ELISA and CIA.

The sensitivity and specificity of CIA in the detection of anti-GBM antibodies were 97.4% and 100%, respectively, compared with 94.9% and 97.9%, respectively, by commercial ELISA. The AUC for CIA and commercial ELISA was 0.987 and 0.966, respectively (Figure 2).

### Precision of the CIA

For the precision testing of the CIA, 2 samples, from 40.2 to 1304** **IU/mL, were tested. For the 2 samples, the max within-run variation was 4.2% and 5.6%, the between-day variation was 6.3% and 4.4%, and the between-run variation was 7.4% and 4.1%, respectively.

## Discussion

Anti-GBM antibodies are a hallmark in the diagnosis of anti-GBM disease.

In recent decades, several novel technologies have been developed for anti-GBM antibody detection, including conventional ELISA and indirect immunofluorescence assay and, more recently, CIA. As ELISAs are only moderately fast, with assay times of approximately 2** **h, the focus has shifted toward a decrease in assay time and ease of use, as well as toward increased sensitivity since several patients were misdiagnosed until a renal biopsy was performed. CIA takes 30 min for the first sample and then 1** **min for the next. Thus, we evaluated the faster CIA method for the detection of anti-GBM antibodies.

The CIA method displayed good agreement with our homemade ELISA coated with recombinant human α3(IV)NC1 expressed in a HEK 293 cell line. Many studies have focused on identifying the epitopes of anti-GBM antibodies. It has been clearly shown that anti-GBM antibodies react with conformational epitopes of α3(IV)NC1, which limits the application of linear synthetic peptides for diagnosis strategies. Accordingly, chimeric molecules of human α3(IV)NC1 and α1(IV)NC1 expressed in a mammalian cell line have been used for epitope mapping [8,9]. However, the human α3(IV)NC1 expressed in the mammalian cell line achieved only low production and was highly expensive for clinical use. Assays using the recombinant antigen expressed in insects were produced, with different performances [6]. The good agreement achieved for CIA and our homemade ELISA may be partly because of the exposure of the target antigen for binding on the beads rather than in a well in the ELISA plate, which might provide more conformational epitope mimicry *in vitro* and expose more cryptic epitopes through the same coating antigen, bovine NC1 alpha 3(IV), than the ELISA plate, which showed better agreement with the clinical diagnosis.

Given the aggressive nature of anti-GBM disease, there is a compelling need for a rapid and sensitive test for the detection and monitoring of anti-GBM antibodies. Plasma exchange is one of the most important therapies for anti-GBM disease. In our study, there were also 2 biopsy-proven anti-GBM disease patients with low levels of anti-GBM antibodies, revealing discrepant results in ELISA assays (Supplementary table 2). Since the indication of cessation is the levels of anti-GBM antibody in plasma exchange, the negative result directed the administration of inadequate regimens.

Only a few studies have evaluated and compared the diagnostic performance of anti-GBM antibody immunoassays. However, we found consistently negative results obtained with all assays for 1 anti-GBM disease patient, which is consistent with the reported prevalence of anti-GBM-negative anti-GBM disease patients [10]. Approximately 2–8% of patients with anti-GBM disease have been reported to be anti-GBM antibody negative by enzyme immunoassays or Western blot [10]. The antibodies of sometimes patients may recognize highly conformational epitopes, which could be found by nonreducing Western blotting, and some may bind to α chains other than α3[5].

This study also had some shortcomings. First, the positivity is unusually low since anti-GBM disease is rare and because this report is a prospective study. Thus, we included another group of patients with biopsy-proven anti-GBM disease to further validate our findings. Second, this report is a single center study, although we have the largest cohort of anti-GBM disease patients in the world. Thus, multicenter studies may be needed.

## Conclusion

The detection of anti-GBM antibodies with CIA demonstrated good sensitivity and specificity and was in good agreement with our homemade ELISA coated with recombinant human α3(IV)NC1, which seems to provide better performance than the commercial ELISA assay in suspected anti-GBM disease patients. The three assays performed in parallel for the diagnosis of anti-GBM disease patients.

## Supplementary Material

Supplemental Material

Supplemental Material
