# Supplementary material for: Comparison of the performance of a chemiluminescence assay and an ELISA for detection of anti-GBM antibodies
Source: Ren Fail. 2019 Dec 29;42(1):48–53. doi: 10.1080/0886022X.2019.1702056 (PMC6968565; doi:10.1080/0886022X.2019.1702056)
Supplement: Supplemental Material [file IRNF_A_1702056_SM8105.docx]

**Table 1** The gene ID and sequence of human NC1 alpha 3(IV) antigen

**Gene ID** 1285

1 cgtggagaca gtggatcacc tgcaacctgg acaacgagag gctttgtctt cacccgacac

61 agtcaaacca cagcaattcc ttcatgtcca gaggggacag tgccactcta cagtgggttt

121 tcttttcttt ttgtacaagg aaatcaacga gcccacggac aagaccttgg aactcttggc

181 agctgcctgc agcgatttac cacaatgcca ttcttattct gcaatgtcaa tgatgtatgt

241 aattttgcat ctcgaaatga ttattcatac tggctgtcaa caccagctct gatgccaatg

301 aacatggctc ccattactgg cagagccctt gagccttata taagcagatg cactgtttgt

361 gaaggtcctg cgatcgccat agccgttcac agccaaacca ctgacattcc tccatgtcct

421 cacggctgga tttctctctg gaaaggattt tcattcatca tgttcacaag tgcaggttct

481 gagggcaccg ggcaagcact ggcctcccct ggctcctgcc tggaagaatt ccgagccagc

541 ccatttctag aatgtcatgg aagaggaacg tgcaactact attcaaattc ctacagtttc

601 tggctggctt cattaaaccc agaaagaatg ttcagaaagc ctattccatc aactgtgaaa

661 gctggggaat tagaaaaaat aataagtcgc tgtcaggtgt gcatgaagaa aagacac
