# Supplementary material for: Comparison of the performance of a chemiluminescence assay and an ELISA for detection of anti-GBM antibodies
Source: Ren Fail. 2019 Dec 29;42(1):48–53. doi: 10.1080/0886022X.2019.1702056 (PMC6968565; doi:10.1080/0886022X.2019.1702056)
Supplement: Supplemental Material [file IRNF_A_1702056_SM8094.docx]

**Table 2 Different results of anti-GBM antibody detection of patients with anti-GBM disease**

| **Patient** | **ELISA (RU/mL)** | **CIA (cu)** |
| --- | --- | --- |
| No.1 | Neg | 30 |
| No.2 | Neg | 24.5 |
